# Manipulation of glycogen and sucrose synthesis increases photosynthetic productivity in cyanobacteria

**DOI:** 10.3389/fmicb.2023.1124274

**Published:** 2023-05-18

**Authors:** Michael Cantrell, Melissa Cano, Jacob Sebesta, Troy Paddock, Wei Xiong, Katherine J. Chou, Jianping Yu

**Affiliations:** Biosciences Center, National Renewable Energy Laboratory, Golden, CO, United States

**Keywords:** photosynthesis, cyanobacteria, glycogen, sucrose, ATP

## Abstract

Photosynthetic productivity is limited by low energy conversion efficiency in naturally evolved photosynthetic organisms, via multiple mechanisms that are not fully understood. Here we show evidence that extends recent findings that cyanobacteria use “futile” cycles in the synthesis and degradation of carbon compounds to dissipate ATP. Reduction of the glycogen cycle or the sucrose cycle in the model cyanobacterium *Synechocystis* 6803 led to redirection of cellular energy toward faster growth under simulated outdoor light conditions in photobioreactors that was accompanied by higher energy charge [concentration ratio of ATP/(ATP + ADP)]. Such manipulation of energy metabolism may have potential in engineering microalgal chassis cells to increase productivity of biomass or target metabolites.

## 1. Introduction

Photosynthesis is nature’s primary CO_2_ capture and utilization (CCU) process, in which solar energy is captured by light harvesting pigments and stored in high-energy metabolites such as ATP and NADPH, which in turn drive CO_2_ fixation and energy storage in carbon-carbon bonds. Production and consumption of high-energy metabolites dictate productivity, thus understanding their management is central to understanding photosynthetic energy conversion process, as well as the rational engineering of microalgal chassis cells for the synthesis of fuels and chemicals.

Since photosynthesis occurs in natural environments with dynamic light, temperature, and nutrient conditions, synthesis and consumption of ATP and NADPH must respond dynamically to changes in the environment. This drives the evolution of an abundance of mechanisms that help balance energy production with demands. This balance can be achieved by modulation of electron transport—either through dissipation by non-photochemical quenching or re-routing of electron transport via cyclic electron transport and alternative electron transport pathways (Allahverdiyeva et al., 2015). Biosynthetic pathways can also act as sinks for excess ATP and NADPH, and can serve to optimize metabolism in response to environmental conditions (Cano et al., 2018).

**FIGURE 1:**
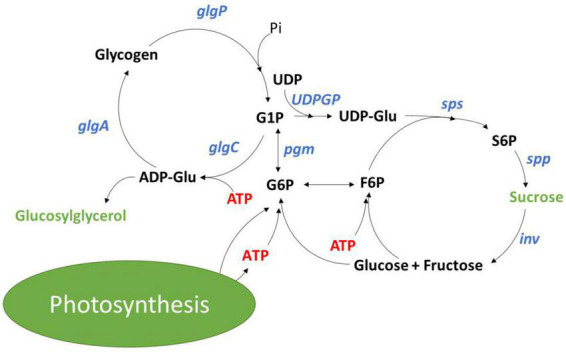
Glycogen and sucrose synthesis cycles in cyanobacteria with metabolites shown in black, enzymes shown in blue and osmolytes shown in green.

Synthesis of carbon reserves, such as glycogen in cyanobacteria as well as starch and lipids in eukaryotic algae, provides a sink for energy during nutrient limitation and serves as a reserve for cellular maintenance at night or during acclimation to changing light and nutrient availability (Vitova et al., 2015; Luan et al., 2019; Ran et al., 2019). In the model cyanobacterium *Synechocystis* sp. PCC 6803 (hereafter referred to as *Synechocystis*), fixed carbon is committed to glycogen synthesis by ADP-glucose pyrophosphorylase (AGPase) encoded by the *glgC* gene (Figure 1; Luan et al., 2019). Previous characterization of a Δ*glgC* mutant that can no longer synthesize glycogen showed that the glycogen synthesis/degradation cycle acts as an energy management mechanism (Cano et al., 2018). Loss of this mechanism leads to a higher energy charge (EC; [ATP]/([ATP + ADP])) in the light, and to the excretion of organic acids under high light, mixotrophic conditions, or nitrogen deprivation (Carrieri et al., 2012; Grundel et al., 2012; Cano et al., 2018). Additionally, blocking glycogen synthesis could provide a means for increasing carbon partitioning into target molecules like mannitol and ethanol (Jacobsen and Frigaard, 2014; Namakoshi et al., 2016). However, a drawback of blocking glycogen synthesis is the reduced fitness under saturating and diurnal light conditions even in the presence of alternative sinks (Suzuki et al., 2010; Grundel et al., 2012).

Sucrose synthesis has been studied regarding its role in protection against increases in salinity. Aquatic environments face dynamic changes in salinity with depth and at mixing zones that drives osmotic loss of water, disrupting ion homeostasis and loss of cellular turgor pressure (Hagemann, 2011). To combat dynamic changes in salinity, cyanobacteria have evolved a suite of mechanisms that can rapidly adjust ion homeostasis. This involves transcriptional reprogramming, effectively increasing active export of Na^+^ and Cl^–^, and triggers the accumulation of osmo-protective organic compounds (Marin et al., 2004; Hagemann, 2011). In response to salt stress, *Synechocystis* accumulates primarily sucrose and glucosylglycerol (Kirsch et al., 2018, 2019). Salt shock is immediately followed by the rapid accumulation of sucrose within the first few hours, which is then followed by the slower accumulation of glucosylglycerol. After 24 h, sucrose content drops, and glucosylglycerol dominates as the primary osmolyte for long term accumulation in response to salt stress (Desplats et al., 2005). Sucrose accumulation is a product of salt ion activation of the sucrose phosphate synthetase (SPS; *sll0045*) and inactivation of a sucrose invertase (INV; *sll0626*) (Hagemann and Marin, 1999; Kirsch et al., 2018). Kirsch et al. (2018) demonstrated that the sucrose synthesis and degradation cycle is also active under non-stressed conditions, with Δ*inv* mutants displaying 10 times the sucrose accumulation as compared to wild-type. To our knowledge, sucrose synthesis has not been studied as an energy management mechanism.

In this study, we show genetic and physiological evidence to support the hypothesis that cyanobacteria use the sucrose synthesis and degradation cycle in addition to the glycogen synthesis and degradation cycle to regulate ATP. In addition, we tested the hypothesis that a *partial inhibition* of glycogen synthesis would improve strain robustness while increasing cellular energy supply toward the synthesis of biomass. Disruption of such energy management mechanisms led to higher cellular energy levels and faster growth under a range of light conditions including simulated outdoor light conditions. These findings suggest a new approach to manipulate cellular energy metabolism toward higher photosynthetic productivity.

## 2. Results

### 2.1. Mutant verification

**FIGURE 2:**
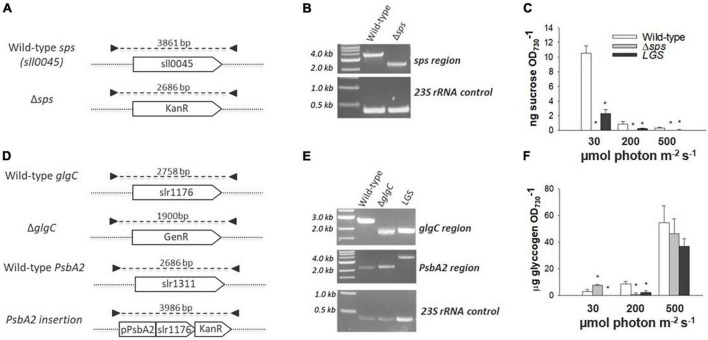
Mutant generation, genotyping by PCR, and phenotype verification. The Δ*sps* mutant was generated by complete removal of the *sps* coding region via homologous recombination with a kanamycin resistance cassette [*KanR*; **(A)**] and homoplasmy was verified by PCR **(B)**. Sucrose content was assayed during exponential growth in cultures acclimated to 30, 200, and 500 μmol photons m^–2^ s^–1^ **(C)**. The low glycogen strain (*LGS*) strain was generated in the Δ*glgC* background (Gentamycin Resistant—*GenR*) by insertion of a *glgC* coding sequence with kanamycin resistance (*KanR*) under the regulation of the native PsbA2 promoter **(D,E)**. Glycogen content was assayed during exponential growth in cultures acclimated to constant 30, 200, and 500 μmol photons m^–2^ s^–1^ **(F)**. The 23S ribosomal RNA was used as an amplification control for each PCR reaction. Symbols (*) represent significant differences from wild-type within light acclimated states based on a one-way ANOVA and Tukey’s HSD *post-hoc* test (*p* < 0.05).

The *sps* gene in *Synechocystis* was completely knocked out by homologous recombination using a kanamycin resistance cassette (Figure 2A). The insertion was verified through PCR (Figure 2B). Complete removal of the *sps* coding sequence led to the loss of sucrose accumulation in exponential cultures acclimated to constant illuminations of 30, 200, and 500 μmol photons m^–2^ s^–1^ (Figure 2C) and after 12 h of salt stress (Supplementary Figure 1). A low glycogen strain (referred to as *LGS*) was generated as previously described in Carrieri et al. (2012). by complementation of a Δ*glgC* mutant, using the *glgC* coding sequence to replace the *psbA2* coding sequence (Figures 2D–E). This strain displayed reduced glycogen levels under constant illuminations of 30, 200, and 500 μmol photons m^–2^ s^–1^ (Figure 2F). This was correlated with lower sucrose concentrations in the *LGS* relative to WT under 30 and 200 μmol photons m^–2^ s^–1^ and after 12 h of incubation with 500 mM salt (Figure 2C and Supplementary Figure 1), indicating a link between the two biosynthetic pathways.

### 2.2. Sucrose or glycogen synthesis mutants exhibit increased energy charge and growth rates under a variety of light conditions

**FIGURE 3:**
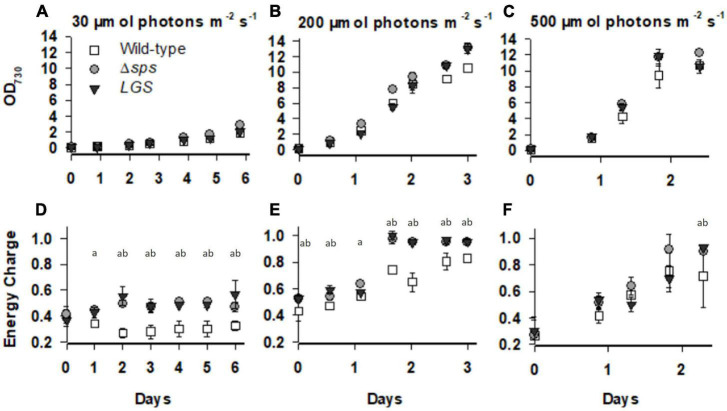
The optical density and energy charge ([ATP]/([ATP] + [ADP])) for wild-type (open squares), Δ*sps* (shaded circles), and *LGS* (black triangles) grown under constant lights intensities of 30 **(A,D)**, 200 **(B,E)**, and 500 **(C,F)** μmol photons m^–2^ s^–1^. Data shown reflects *n* = 3–5 cultures with error bars reflecting the standard deviation. Letters denote significant differences based on a one-way ANOVA (*P* < 0.05) and Tukey’s HSD *post-hoc* test with (a) denoting significant differences between wild-type and Δ*sps* and (b) denoting significant differences between wild-type and *LGS*.

We hypothesized, based on prior observations on the Δ*glgC* mutant, that each strain may display higher ATP levels which may lead to faster growth under photoautotrophic conditions. The mutants were grown under constant light with 5% CO_2_ in shake flask conditions, and under sinusoidal light in the NREL’s lab-built Simulated Algal Growth Environment (SAGE) bioreactors, which are capable of supporting algal growth in 2-L bottles while mimicking outdoor light and temperature conditions (Supplementary Figure 2A). Intracellular ATP and ADP levels were assessed using a fast luciferase-based assay as described in Cano et al. (2018). Under a continuous light of 30 μmol photons m^–2^ s^–1^, we observed increases in growth rate for Δ*sps* vs. wild-type (11.3%; Table 1) and found that both Δ*sps* and *LGS* displayed significant increases in energy charge ([ATP]/[ATP + ADP]; Figure 3D). Under a continuous 200 μmol photons m^–2^ s^–1^ Δ*sps* and *LGS* exponential growth rates were 18.7% (*p* < 0.05) and 8.7% faster than wild-type (Figure 3B and Table 1), and had significantly higher energy charge (Figure 3E). Under 500 μmol photons m^–2^ s^–1^, each strain exhibited reduced growth rates relative to 200 μmol photons m^–2^ s^–1^ and energy charge was significantly increased only during early stationary phase in mutants (Figure 3F). Under these saturating light conditions, only the *LGS* displayed significantly faster growth than wild-type (6.8%, *p* < 0.05).

**TABLE 1:** Effects of acclimation state on mutant exponential growth rate and pigment content for shake flask cultures grown under constant lights intensities of 30, 200, and 500 μmol photons m^–2^ s^–1^.

30 μmol photons m^–2^ s^–1^				
	**Units**	**Wild-type**	**Δ*sps***	***LGS***
Growth rate	Day**^–^**^1^	0.53 ± 0.02	0.59 ± 0.03*	0.54 ± 0.01
Chlorophyll *a*	μg OD**^–^**^1^	1.00 ± 0.06	0.64 ± 0.02*	0.79 ± 0.12*
Carotenoids	μg OD**^–^**^1^	0.31 ± 0.03	0.31 ± 0.01	0.32 ± 0.02
**200 μmol photons m^–^^2^ s^–^^1^**				
	**Units**	**Wild-type**	**Δ*sps***	***LGS***
Growth rate	Day**^–^**^1^	2.68 ± 0.22	3.18 ± 0.03*	2.91 ± 0.01
Chlorophyll *a*	μg OD**^–^**^1^	1.01 ± 0.20	0.65 ± 0.17	0.74 ± 0.01
Carotenoids	μg OD**^–^**^1^	0.29 ± 0.02	0.31 ± 0.03	0.34 ± 0.03
**500 μmol photons m^–^^2^ s^–^^1^**				
	**Units**	**Wild-type**	**Δ*sps***	***LGS***
Growth rate	Day**^–^**^1^	2.50 ± 0.03	2.62 ± 0.07	2.67 ± 0.04*
Chlorophyll *a*	μg OD**^–^**^1^	0.45 ± 0.03	0.45 ± 0.04	0.5 ± 0.08
Carotenoids	μg OD**^–^**^1^	0.40 ± 0.02	0.43 ± 0.06	0.38 ± 0.06

Data represents the mean ± s.d. (*n* = 3–4). Symbols (*) represent significant differences from wild-type within light acclimated states based on a one-way ANOVA and Tukey’s HSD *post-hoc* test (*p* < 0.05).

### 2.3. Increased growth rates are correlated with high photosynthetic rates during growth in moderate/high light

**FIGURE 4:**
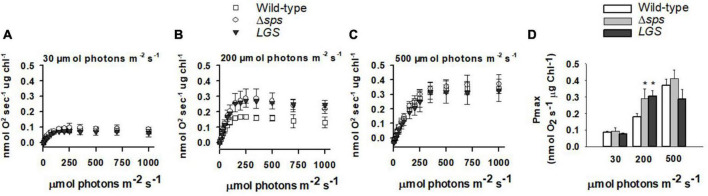
Photosynthesis vs. irradiance for wild-type (open squares), Δ*sps* (shaded circles), and *LGS* (black triangles) grown under constant lights intensities of 30 **(A)**, 200 **(B)**, and 500 μmol photons m^–2^ s^–1^ **(C)** and maximum photosynthetic rate (Pmax) for each culture condition **(D)**. Values displayed reflect oxygen evolution rates (nmol O_2_ s^–1^ μg chl^–1^) for samples exposed to sequentially increasing light intensities. Data shown reflects *n* = 3 with error bars reflecting the standard deviation. Symbols (*) represent significant differences from wild-type within light acclimated states based on a one-way ANOVA and Tukey’s HSD *post-hoc* test (*p* < 0.05).

We investigated the impact of each genetic modification on photosynthesis by measuring photosynthesis vs. irradiances (PI curve) with sequential 60 s light steps supplemented with 10 mM sodium bicarbonate. Across the constant light conditions investigated, we found that the maximum photosynthetic rate (Pmax; Figure 4) and saturation point of photosynthesis (IK) increased with culture light intensity (Supplementary Figure 3). When comparing between strains, we found that only mutants grown under 200 μmol photons m^–2^ s^–1^ displayed a significant increase in Pmax (Figures 4B, D). Under these conditions both Δ*sps* and *LGS* displayed a 61–69% increase in Pmax with no significant differences observed between light limited slope (α) and IK (e).

### 2.4. Mutants show increases in the accumulation rates of ash free dry weight (AFDW) in SAGE reactors and a higher energy charge at dawn

**FIGURE 5:**
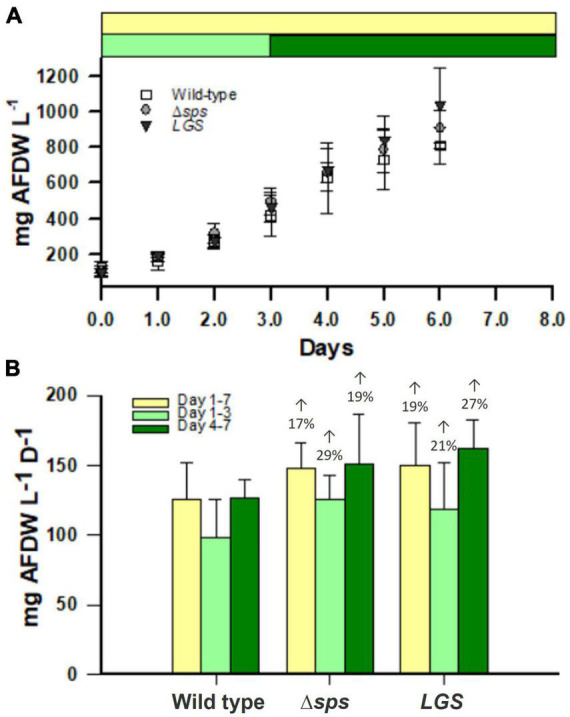
Ash free dry weight (AFDW) accumulation in SAGE reactors **(A)** for wild-type (open squares), Δ*sps* (shaded circles), and *LGS* (black triangles). Accumulation rates are shown for SAGE reactors **(B)** for the entire growth period (yellow), the beginning of the growth period (light green) and the end of the growth period (dark green). Data shown reflects *n* = 4 cultures with error bars reflecting the standard deviation. Percentages provided above bars in **(B)** denote significant differences from wild-type based on a one-way ANOVA and Tukey’s HSD *post-hoc* test (*P* < 0.05).

To determine if this improved growth is conserved under outdoor light conditions, we investigated productivity based on AFDW accumulation in the NREL simulated algae growth environment (SAGE) reactors. These were operated at a constant 30 °C under light conditions simulating those found during the fall at the Arizona Center for Algae Technology and Innovation (AZCATI) test site, which followed a sinusoidal light rhythm with intensities peaking at 1,700 photons m^–2^ s^–1^ (Supplementary Figures 2A, B). AFDW was assessed daily at solar dawn for cultures after 3 days of pre-acclimation under this light regime. We found that Δ*sps* and *LGS* displayed increases in AFDW accumulation of 17 and 19%, respectively (Figure 5). In the first 3 days in particular, the sps mutant grew much faster with a biomass accumulation rate 29% higher than the wild type.

**FIGURE 6:**
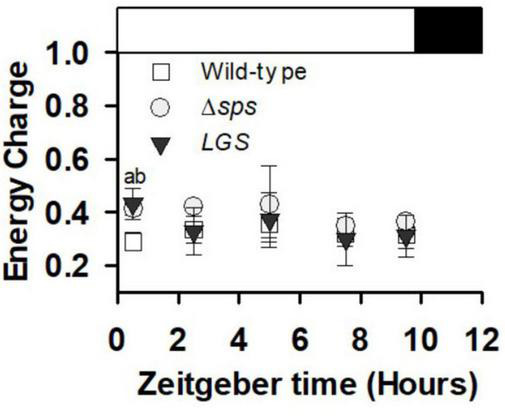
Energy charge ([ATP]/([ATP] + [ADP])) collected during early exponential growth (day 3) for cultures grown under sinusoidal light in SAGE reactors. Data shown reflects *n* = 3–5 with error bars reflecting the standard deviation. Letters denote significant differences based on a one-way ANOVA (*P* < 0.05) and Tukey’s HSD *post hoc* test with (a) denoting significant differences between wild-type and Δsps and (b) denoting significant differences between wild-type and *LGS*.

In addition to a high rate of AFDW accumulation, we found that under sinusoidal light conditions cultures maintained a significantly higher energy charge 30 min after dawn (at Zeitgeber time 0.5 h) (Figure 6).

## 3. Discussion

Understanding the regulation of high energy molecules such as ATP and NADPH is central in understanding biological processes such as photosynthesis, and in engineering microalgal chassis cells for carbon sequestration and utilization. This work expanded our prior findings on mechanisms for intracellular energy management in *Synechocystis*. Previous work demonstrated a high energy charge in a mutant that can no longer accumulate glycogen (Δ*glgC*). However, this strain had reduced fitness under saturating light and diurnal conditions (Grundel et al., 2012; Cano et al., 2018). Therefore, we hypothesized that a strain with low glycogen content may display high EC and improved photosynthetic rates without reducing fitness. Here we found that the complemented Δ*glgC* mutant strain [*LGS* (Carrieri et al., 2012)] displayed increased EC as well as growth rates.

Glycogen plays an essential role in both balancing energy homeostasis and initiation of photosynthetic metabolism. Shinde et al. (2020) demonstrated that glycogen degradation is required for initiating photosynthesis upon transition from dark to light conditions. Furthermore, they showed that absence of glycogen synthesis in the Δ*glgC* mutant or the oxidative pentose phosphate pathway in the Δ*gnd* mutant led to reduced photosynthetic efficiency and slower PSI (P700^+^) re-reduction rates. This was used to support the hypothesis that glycogen degradation through the OPP pathway is essential for initiation of photosynthesis by generating the NADPH needed to activate key Calvin-Benson cycle enzymes (Shinde et al., 2020). We previously showed that the Δ*glgC* mutant under photoautotrophic conditions experiences metabolite overflow, with both increased EC and organic acid excretion to the extracellular medium (Carrieri et al., 2012, 2015; Cano et al., 2018). Taken together, these results indicate that glycogen synthesis and degradation facilitate metabolic homeostasis by either generating NADPH when reducing energy is low or can act as a sink for carbon and ATP when energy is in excess. Here we found that faster growth in our LGS was correlated with higher energy charge (Figures 3, 6) and a lower glycogen content (Figure 2F and Supplementary Figure 4). In contrast to moderate and diurnal light levels, our LGS displayed comparable growth rates and glycogen levels to wild-type under constant saturating light intensities (500 μmol photon m^–2^ s^–1^), reflecting light induction of the *psbA2* promoter used for complementation. This suggests that even under non-stress conditions, cyanobacteria use a glycogen synthesis/degradation “futile cycle” to help maintain energy balance and that reducing glycogen storage under non-saturating light conditions can enhance biomass productivity.

Sucrose synthesis is essential for adaption to salt stress. Previous characterization of the Δ*sps* mutant demonstrated its importance for growth under salt stress, with Δ*sps* displaying lower growth in BG-11 supplemented with 684 mM NaCl. In the wild-type, despite a consistent growth reduction, 684 mM NaCl leads to a transient sucrose accumulation that peaks at 4 h and then drops off significantly after 12 h (Desplats et al., 2005). This decrease of sucrose levels reflects the activity of invertase, with the *Synechocystis* invertase mutant displaying no drop off in sucrose under salt stress and ∼9 times the sucrose concentration in standard BG-11 (Kirsch et al., 2018). Here we demonstrate that without inducing salt stress (growth in normal BG-11), the Δ*sps* mutant displays increased EC and improved growth under continuous moderate light and simulated outdoor light conditions (diurnal sinusoidal fluctuating light with high maximum intensity) (Figures 3, 5, 6). The higher EC in this case reflects the savings of two ATP per sucrose synthesis cycle and indicates that the sucrose cycle plays a role in energy regulation under constant light conditions. The observations of higher EC and faster growth in the Δ*sps* mutant indicate that faster growth is not just a consequence of carbon redirection, as there is minimal sucrose accumulation under these conditions.

Sucrose and glycogen synthesis reflect metabolically linked cycles and their reduction increases fitness under moderate light. Previous efforts to modify carbon partitioning in cyanobacteria have highlighted the interconnection of the sucrose and glycogen cycles. Sucrose and glycogen synthesis both involve glucose-6-phosphate generated from the Calvin Benson cycle or through regeneration of glucose through glycogen or sucrose catabolism. Sucrose and glycogen pools appear linked, though flux between them is dependent on environmental conditions. Under salt stress, knockdown of glycogen synthesis (*glgC*) limited sucrose production in *Synechococcus* PCC7942, indicating that under environmental stress glycogen acts as a carbon pool for sucrose synthesis (Qiao et al., 2018). This is supported by the observations that overexpression of both *glgC* and *sps* can significantly increase sucrose accumulation, and by similar observations on the dependence of sucrose synthesis on the glycogen pool in *Synechococcus* UTEX 2973 under salt stress (Qiao et al., 2018; Lin et al., 2020). In contrast, under non-salt stress conditions, deletion of *glgC* in *Synechococcus* PCC7942 was shown to increase sucrose accumulation rates by 10–15% (Ducat et al., 2012). This suggests that under normal conditions, sucrose, glycogen synthesis and anabolic metabolism all compete for glucose-6-phosphate. We found that either knocking out sucrose synthesis or reducing glycogen synthesis led to similar phenotypes. While both appears to be impacted in their acclimation state based on their chlorophyll content under low and moderate constant light, each mutant displayed increases in growth rates, especially under diurnal conditions (Figure 5). These increases were correlated with a high EC, which may also facilitate improved photosynthetic function observed at constant irradiances of 200 μmol photons m^–2^ s^–1^ (Figures 3, 4).

## 4. Materials and methods

### 4.1. Growth conditions

Wild-type *Synechocystis* sp. PCC6803, the *LGS*, and Δ*sps* strains were grown photoautotrophically in standard BG-11 medium supplemented with 100 mM NaHCO_3_, 20 mM TES (pH 8) in 250 ml flasks unless otherwise noted. The *LGS* strain was constructed as previously described in Carrieri et al. (2012) and the Δ*sps* mutant was constructed by homologous recombination with the pIDTSMART vector containing a kanamycin cassette flanked by *sps* 5′ and 3′ regions. Segregation of the Δ*sps* mutant was verified by PCR product analysis. Δ*sps* and the *LGS* were maintained on plates of BG-11 with 25 μg ml^–1^ kanamycin or 25 μg ml^–1^ kanamycin and 25 μg ml^–1^ gentamycin, respectively. For shake flask experiments, liquid cultures were inoculated from plates and allowed to acclimate to the experimental conditions used for 3–6 days with dilution every 2–3 days to maintain cultures in exponential growth.

Shake flask cultures were grown in a Percival growth chamber maintained at 30°C with 5% ambient CO_2_ under each respective light conditions. Moderate light intensities (30, 200, and 500 μmol photons m^–2^ s^–1^) were provided by cool light fluorescent lamps. Culture growth and dilution was performed using absorbance measurements at 730 nm (OD_730 nm_) with a Beckman Coulter DU 900 spectrometer. All experiments used cultures inoculated at an OD_730 nm_ of 0.09–0.10 from an exponentially grown culture—tested to be axenic by plating on LB medium. Exponential growth rates were calculated by linear regression using the exponential growth equation $\mu =\frac{\ln\left(F\right)-\text{ln}\,\left(I\right)}{\Delta \,t}$ where *F* is the final cell concentration, *I* the initial cell concentration, μ is the specific growth rate (day^–1^) and *t* time in days.

SAGE reactor experiments were maintained at 30°C by water bath and illuminated using light panels (Renology, Ontario, CA, USA). We programmed this light panel to mimic daily light patterns recorded at the Arizona Center for Algae Technology and Innovation (AZCATI) during a week in September. This generally followed a 12:12 light: dark sinusoidal light cycle with peak light intensities of 1554 μmol photons m^–2^ s^–1^ (Supplementary Figure 2B).

### 4.2. ATP and ADP determination

ATP and ADP determination was performed as described in Cano et al. (2018) using the ATP/ADP ratio assay kit (Sigma Aldrich, cat. no. MAK135). In short, 10 μl culture was sampled from each flask directly into 96 well plate containing 90 μl sigma cell lysis reagent. Luminescence was measured as described by the manufacturer using a Tecan Infinite M200 Pro luminometer. Energy charge as shown throughout the manuscript reflects the concentration [ATP]/([ATP] + [ADP]).

### 4.3. Glycogen quantification

Glycogen quantification was performed as described in Vidal and Venegas-Caleron (2019). In short, 10 ml of sample (2–3 OD units) was collected by repeated centrifugation at 10,000 × *g* at room temperature (about 23°C) in a 2 ml Eppendorf tube, decanted and immediately frozen. Alkaline thermolysis was performed by boiling in 30% KOH for 90 min at 95°C in a dry bath. Glycogen was then precipitated from the raw extract with 2 ml of cold, 100% ethanol at −80°C overnight. Precipitated glycogen was then collected by centrifugation at 16,000 × *g* for 30 min at 4°C. Isolated glycogen was digested to glucose by incubation with 10 units of aminoglucosidase (Sigma Aldrich, cat. no. A7095) at 55°C for 2 h. Glucose quantification was then performed on dilutions using the glucose assay kit (Sigma Aldrich, cat. no. GAGO-20) (Vidal and Venegas-Caleron, 2019).

### 4.4. Sucrose quantification

Sucrose quantification was performed as described in Kirsch et al. (2019) with the following modifications. Samples were harvested by centrifugation at 10,000 × *g* at room temperature in a 2 ml Eppendorf tube, decanted and immediately frozen. Pelleted samples were resuspended in 80% ethanol and incubated overnight at 65°C. After centrifugation the supernatants were collected and dried by a speedvac concentrator (Savant ISS110). Dried samples were resuspended in diH_2_O and used for sucrose quantification using a Sucrose quantification kit (Sigma Aldrich, cat. no. MAK267) following the manufacturer’s instructions (Kirsch et al., 2019).

### 4.5. Dry weight and productivity determination

Dry weights were determined for 10 ml samples filtered onto pre-washed Whatman glass microfiber filters (Fisher cat. no. 18-250-70) in Aluminum Tins (Fisher cat. no. 08-732-102). Samples were initially dried in an oven at 55°C and then allowed to come to room temperature before being weighed to obtain the total suspended solids. Ash content of samples was subsequently determined by burning dry samples in a furnace at 575°C for 180 min. Samples were allowed to cool for 30 min prior to weight determination of the ash content of samples. AFDW was determined by subtracting the ash content from the total suspended solids and was normalized by volume for calculations of AFDW accumulation.

### 4.6. Chlorophyll and total carotenoid quantification

Pigments were extracted in 100% methanol overnight and spectra was collected using a Beckman coulter DU 900 spectrometer. Samples were pelleted by centrifugation at 17,000 × *g* for 10 min with 0.01% (v/v) Tween 20. Pellets generated were dissolved in methanol overnight at 4°C and centrifuged at 17,000 × *g* for 1 min to clarify solutions prior to spectra collection. Chl *a* and total carotenoid content were determined as previously described (Wellburn, 1994; Ritchie, 2006).

### 4.7. Photosynthesis vs. irradiance curves

Rapid light curves were generated using a Clark electrode and 1 min light steps. Samples were harvested and immediately transferred to the Clark electrode cuvette (3–6 μg Chlorophyll *a*/ml) with 10 mM sodium bicarbonate and dark incubated at 30°C for 10 min. After dark incubation, cells were exposed to sequential 1 min light levels of 5, 15, 25, 35, 50, 75, 100, 150, 200, 250, 350, 500, 700, and 1000 μmol photon m^–2^ s^–1^. P vs. I curves were fit using the exponential difference equation using the curve fitting tool described by Ritchie (2008) to derive the maximum oxygen evolution rate (Pmax), the light limited slope (α) and the irradiance at saturation (IK) (Ritchie, 2008).

### 4.8. Statistics

All measurements were made on at least three independent cultures. Statistically significant differences were assessed by one-way ANOVA using Tukey’s honestly significant differences test (Tukey’s HSD) to delineate between significantly different groups.

## Data availability statement

The raw data supporting the conclusions of this article will be made available by the authors, without undue reservation.

## Author contributions

MiC and JY designed the study. MiC, TP, and MeC collected the data. MiC, WX, KC, and JY analyzed the data. MiC drafted the manuscript. All authors edited manuscript and agreed on the submitted manuscript.

## Author disclaimer

The views expressed in the article do not necessarily represent the views of the DOE or the U.S. Government. The U.S. Government retains and the publisher, by accepting the article for publication, acknowledges that the U.S. Government retains a non-exclusive, paid-up, irrevocable, worldwide license to publish or reproduce the published form of this work, or allow others to do so, for U.S. Government purposes.

## Supplementary material

Supplementary Figure 1 Sucrose accumulation in mutants after 12 h of salt stress under 30 μmol photons m^–2^ s^–1^. Symbols (*) denote significant differences based on a one-way ANOVA (*P* < 0.05) from wild-type. Data shown reflects *n* = 3 with error bars reflecting the standard deviation.

Supplementary Figure 2 SAGE reactor culture conditions and OD_730_ collected for each experiment. **(A)** Image of SAGE reactor culture conditions. **(B)** Light regime provided to SAGE reactor cultures based on irradiances collected at the AZCATI cultivation site. **(C)** OD_730_ measurements for culture growth with 3 days of pre-acclimation.

Supplementary Figure 3 Light limited slope (α) and irradiance at saturation (IK) for P vs. I curves under different constant light culture conditions. Data shown reflects *n* = 3–5 cultures with error bars reflecting the standard deviation.

Supplementary Figure 4 Glycogen quantification for SAGE reactor cultures. Data shown reflects *n* = 3–5 cultures with error bars reflecting the standard deviation. Letters denote significant differences based on a one-way ANOVA (*P* < 0.05) and Tukey’s HSD *post-hoc* test with (a) denoting significant differences between wild-type and Δ*sps* and (b) denoting significant differences between wild-type and *LGS*.
