# Supplementary figures and images for: Manipulation of glycogen and sucrose synthesis increases photosynthetic productivity in cyanobacteria
Source: Front Microbiol. 2023 May 18;14:1124274. doi: 10.3389/fmicb.2023.1124274 (PMC10233058; doi:10.3389/fmicb.2023.1124274)

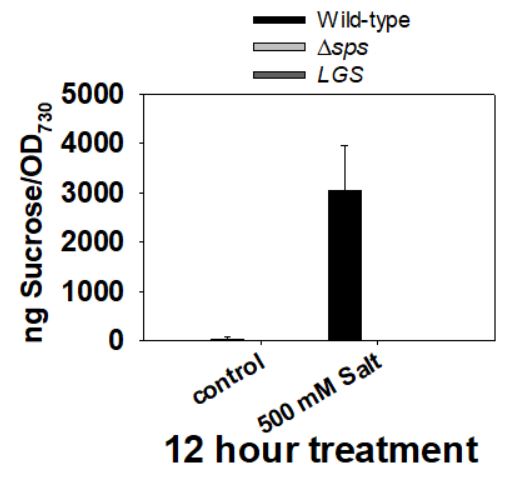

Supplement: Supplementary Figure 1 — Sucrose accumulation in mutants after 12 h of salt stress under 30 μmol photons m–2 s–1. Symbols (*) denote significant differences based on a one-way ANOVA (P < 0.05) from wild-type. Data shown reflects n = 3 with error bars reflecting the standard deviation. [file Image_1.jpeg]

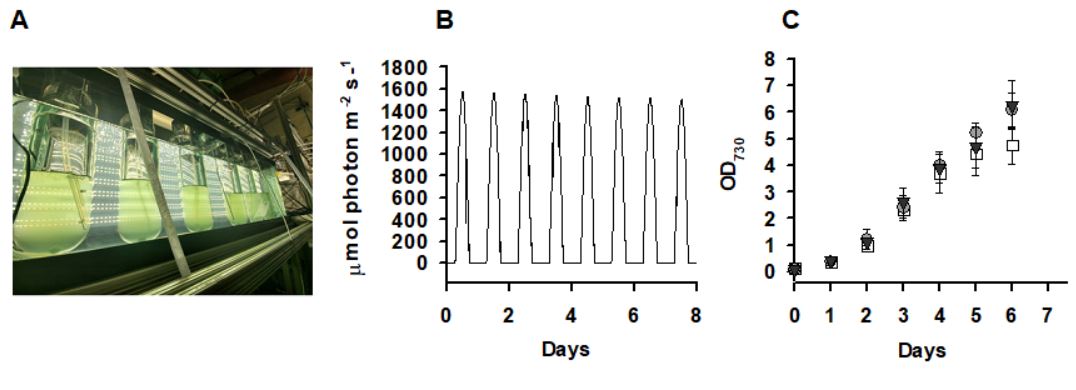

Supplement: Supplementary Figure 2 — SAGE reactor culture conditions and OD730 collected for each experiment. (A) Image of SAGE reactor culture conditions. (B) Light regime provided to SAGE reactor cultures based on irradiances collected at the AZCATI cultivation site. (C) OD730 measurements for culture growth with 3 days of pre-acclimation. [file Image_2.jpeg]

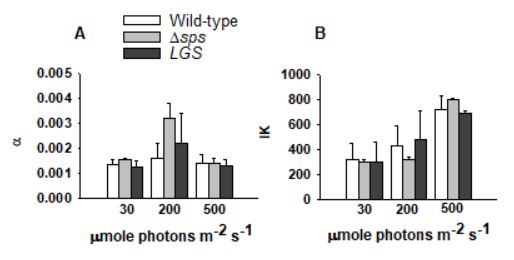

Supplement: Supplementary Figure 3 — Light limited slope (α) and irradiance at saturation (IK) for P vs. I curves under different constant light culture conditions. Data shown reflects n = 3–5 cultures with error bars reflecting the standard deviation. [file Image_3.jpeg]

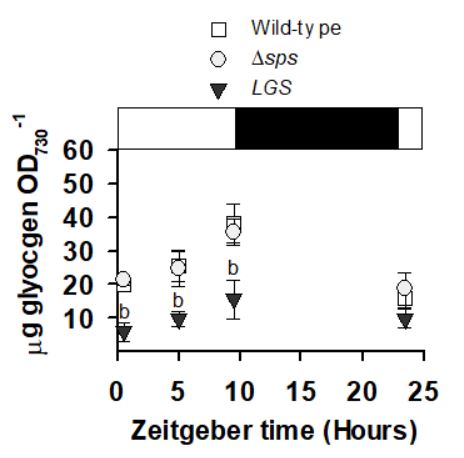

Supplement: Supplementary Figure 4 — Glycogen quantification for SAGE reactor cultures. Data shown reflects n = 3–5 cultures with error bars reflecting the standard deviation. Letters denote significant differences based on a one-way ANOVA (P < 0.05) and Tukey’s HSD post-hoc test with (a) denoting significant differences between wild-type and Δsps and (b) denoting significant differences between wild-type and LGS. [file Image_4.jpeg]
